# The function of BCL9 in Wnt/β-catenin signaling and colorectal cancer cells

**DOI:** 10.1186/1471-2407-8-199

**Published:** 2008-07-15

**Authors:** Marc de la Roche, Jesper Worm, Mariann Bienz

**Affiliations:** 1 MRC Laboratory of Molecular Biology, Hills Road, Cambridge CB2 0QH, UK; 2 Santaris Pharma, Bøge Allé 3, DK-2970 Hørsholm, Denmark

## Abstract

**Background:**

Most cases of colorectal cancer are initiated by hyperactivation of the Wnt/β-catenin pathway due to mutations in the APC tumour suppressor, or in β-catenin itself. A recently discovered component of this pathway is Legless, which is essential for Wnt-induced transcription during *Drosophila *development. Limited functional information is available for its two mammalian relatives, BCL9 and B9L/BCL9-2: like Legless, these proteins bind to β-catenin, and RNAi-mediated depletion of B9L/BCL9-2 has revealed that this protein is required for efficient β-catenin-mediated transcription in mammalian cell lines. No loss-of-function data are available for BCL9.

**Methods:**

We have used overexpression of dominant-negative forms of BCL9, and RNAi-mediated depletion, to study its function in human cell lines with elevated Wnt pathway activity, including colorectal cancer cells.

**Results:**

We found that BCL9 is required for efficient β-catenin-mediated transcription in Wnt-stimulated HEK 293 cells, and in the SW480 colorectal cancer cell line whose Wnt pathway is active due to *APC *mutation. Dominant-negative mutants of BCL9 indicated that its function depends not only on its β-catenin ligand, but also on an unknown ligand of its C-terminus. Finally, we show that *BCL9 *and *B9L *are both Wnt-inducible genes, hyperexpressed in colorectal cancer cell lines, indicating that they are part of a positive feedback loop.

**Conclusion:**

BCL9 is required for efficient β-catenin-mediated transcription in human cell lines whose Wnt pathway is active, including colorectal cancer cells, indicating its potential as a drug target in colorectal cancer.

## Background

The canonical Wnt signaling pathway changes the transcriptional program of cells, and controls genes with important functions during normal and malignant development [1-3]. A key effector of this pathway is β-catenin, which is normally phosphorylated and targeted for degradation by the Axin complex that also contains the Adenomatous polyposis coli (APC) tumor suppressor. This complex is inhibited in response to Wnt stimulation, allowing unphosphorylated β-catenin to accumulate and bind to the nuclear TCF/LEF factors to stimulate the transcription of Wnt target genes. This trans-activation function of β-catenin involves the recruitment of a range of different co-factors that bind to its C-terminus, including chromatin modifying and remodelling factors such as CBP, Brg-1 and SET1 [4-10], TATA-binding protein and associated factors [11,12], and also a transcriptional elongation factor [13]. Well-established transcriptional target genes of this pathway include *c-myc*, *AXIN2 *and *CD44*, whose expression is upregulated in a TCF-dependent fashion in intestinal crypts, and in colorectal neoplasias [14-19].

Recently, two new nuclear components of the canonical Wnt pathway have been discovered in *Drosophila*, called Pygopus (Pygo) and Legless (Lgs), which are required for the transcriptional activity of Armadillo (the *Drosophila *β-catenin) [20-23]. Each of these components has two counterparts in mammals (Pygo1 and Pygo2, BCL9 and BCL9-2/B9L, respectively, whereby BCL9-2 refers to the murine, and B9L to the human ortholog; below, we shall either name these proteins individually, or refer to them collectively as Pygo and BCL9 proteins); siRNA depletion experiments have indicated a role of Pygo1 and Pygo2, and of BCL9-2/B9L for efficient TCF-mediated transcription in colorectal cancer cells [21,24,25]. Likewise, in vertebrate tissues, Pygo1 and Pygo2 contribute to TCF-mediated transcription [26-29]. Nothing is known as yet about the function of BCL9 proteins during vertebrate development.

Lgs/BCL9 proteins are adaptors between Armadillo/β-catenin and Pygo proteins [20,30], and the interactions of Lgs with these binding partners are critical for normal development in flies [31,32]. The molecular roles of Pygo and Lgs/BCL9 in the Wnt pathway are currently debated: these proteins could act to recruit an additional transcriptional co-factor that synergizes with the Armadillo/β-catenin co-factors during the transcription of TCF target genes [13,20,30,33], or they could capture nuclear Armadillo/β-catenin to facilitate its recruitment to TCF target genes [34,35].

Given their activity in colorectal cancer cells [21,24,25], Pygo proteins and B9L may provide new targets for Wnt signaling inhibitors. The latter are particularly promising since there is evidence that BCL9-2/B9L may predispose epithelial cells towards a mesenchymal fate [25]. However, there is very little functional information on BCL9. We thus set out to study the function of this protein in human cell lines whose Wnt pathway is active.

## Methods

### Plasmids

A pCDNA3.1 vector encoding FLAG-tagged human BCL9 was kindly provided by H. Clevers. pCDNA3.1 encoding FLAG-tagged mouse BCL9-2 [25], B9L or B9LΔCter [24] have been described; GFP-tagged B9L [24] was also used. HD2 of human BCL9 (amino acids 343–396) was subcloned into the bacterial expression vector pOPTX (containing an N-terminal thioredoxin tag; kindly provided by R. Williams). The P348G, L351A, Q355E, HRE358-360AKQ, L363F, L366K, L373A and E377Q point mutations were introduced into the BCL9 HD2 domain with the QuickChange mutagenesis kit (Stratagene), and each construct was confirmed by sequencing. To delete the Pygo binding site (ΔHD1) in BCL9-2, an internal deletion of amino acids 238–265 (inclusive) was generated by PCR-mediated mutagenesis. Similarly, for BCL9ΔC, a stop codon was inserted into FLAG-BCL9 after amino acid 849. For the photobleaching experiments, a BCL9 fragment spanning HD1 and HD2 (HD1+2, amino acids 1–727) was subcloned into pEGFP-C1 (Clontech) using the BamHI restriction sites. HA-tagged β-catenin was kindly provided by A. Ben-Ze'ev [36].

### *In vitro *binding assays

^35^S-labeled proteins were generated by *in vitro *transcription/translation using T7 polymerase (Promega). For GST-fusion pull-down assays, glutathione beads containing 10 μg of GST or GST-ARD (GST fused to amino acids 134–671 of β-catenin [37]) were incubated with *in vitro *translated ^35^S-labeled wild-type (wt) or mutant HD2 domains in PBS containing 0.1% Tween-20 and 1% BSA (PBS-TB) for 4 hours at 5°C. Beads with bound proteins were washed five times in PBS-TB, once in PBS and subsequently resolved by SDS-PAGE prior to autoradiography.

### Cell transfections and transcription assays

HEK 293, HEK 293T and HCT116 cells were grown in Dulbecco's modified Eagles medium (DMEM) while SW480 cells were grown in Leibowitz L-15 medium. All media was supplemented with 10% fetal calf serum. Wnt pathway activity was induced by treating cells with 20 mM LiCl (or NaCl, as control) or conditioned media obtained from L-cells expressing Wnt3A for 6 hours. Cells were transfected with the Lipofectamine 2000 transfection reagent according to Manufacturer's protocol (Invitrogen). siRNAs against human BCL9, BCL9-2 and β-catenin were obtained from Dharmacon; 20 or 80 nmol of 4 pooled siRNAs were transfected per well (of 24-well or 6-well plates, respectively). Co-immunoprecipitation or quantitative RT-PCR (RT-qPCR) experiments were carried using cells grown in 6-well plates and transfected with 1 μg per well of each plasmid or 80 pmols of siRNAs. For co-immuno-precipitations, HEK 293 cells were harvested and lysed 24 hours after transfection; mouse α-FLAG monoclonal antibody M2 (Sigma Aldrich) was used for immunoprecipitation. Cells for RT-qPCR were harvested 24- and 48-hours post transfection (with plasmids or siRNAs), and RNA was isolated using the TRIZOL reagent (Invitrogen) according to the manufacturer's protocol.

For immunofluorescence, 250 ng of each plasmid was used to transfect cells grown on 13 mm coverslips in 24-well plates. SW480 and HCT116 cells were fixed and stained with rabbit α-FLAG (Sigma Aldrich) or mouse monoclonal α-β-catenin C19220 (BD Transduction Laboratories) antibodies 24 hours after transfection as described [38]. For TOPFLASH reporter assays, 250 ng of each tester plasmid, or 20 pmols of each siRNA, per well were used to transfect cells (in 24-well plates), in addition to 100 ng of TOPFLASH or FOPFLASH plasmids and 100 ng thymidine kinase (TK)-renilla plasmid (as internal control). TOPFLASH assays were performed using the Dual-Luciferase Reporter Assay System (Promega) as previously described [38,39]. Relative luciferase values were obtained from triplicate samples (from 2–4 independent experiments) by dividing the firefly luciferase values (from TOPFLASH or FOPFLASH) by the renilla luciferase values (from TK-renilla), and standard deviations were calculated. Paired T-tests were performed to assess statistical significance where the observed differences were small.

### RT-qPCR

cDNA was synthesized from total RNA (2.5 μg) primed with random hexamers and Superscript III (Invitrogen). RT-qPCR reactions were carried out with the ABI7900 Taqman thermocycler (Applied Biosystems). Reaction mixtures for amplification (10 μl) contained 0.5 μl of each gene expression assay (AB Biotech), 5 μl of 2 × Master Mix (AB Biotech) 1 μl of cDNA (~50 ng) and water to 10 μl. The following gene expression assays (from Applied Biosystems) were used: *B9L *(Hs00699441), *HPRT1 *(Hs99999909), *AXIN2 *(Hs00610344), *CD44 *(Hs00153304), and *β-Actin *(Hs99999903). Additional gene target primer and probe sets were designed with the GenScript qPCR primer design software, and used as follows (all sequences of primers and probes, respectively, are 5' to 3'): *c-myc*, FAM-AAGACAGCGGCAGCCCGAAC-TAMRA, TCAGAGAAGCTGGCCTCCTA and AGGTACAAGCTGGAGGTGGA; TATA-box binding protein (*TBP*), FAM-TACCGCAGCAAACCGCTTGG-TAMRA, AAAGACCATTGCACTTC GTG and GGTTCGTGGCTCTCTTATCC; *BCL9*, FAM-CGATTATTCCATCTGAGAAGC CCA-TAMRA, CCACCGGAATACCTGAGTTT and AGGGAAATATTGCAGCGTCT. Primers and probes were used at a final concentration of 0.2 μM and 0.5 μM respectively. Values for gene expression in each case were calculated relative to a standard curve of *TBP *expression whose consistency from different samples was confirmed in each case using *HPRT1 *and *β-Actin *expression. Comparative qPCR revealed that the levels of *BCL9 *and *B9L *transcripts in transfected HEK 293 cells were 45× and 90×, respectively, above those of endogenous genes.

### Immunoblotting

Cell lysates were prepared in lysis buffer consisting of 50 mM Tris-HCl pH 7.4, 1% NP-40, 150 mM NaCl and 1 mM MgCl_2 _and a mixture of protease inhibitors. Total protein content was determined with Coomassie Plus Protein Assay Reagent (Pierce). Lysates corresponding to 100 μg of total protein were analysed by PAGE with 3–8% gradient Tris-acetate gels (Invitrogen). Proteins were transferred to a PVDF membrane in transfer buffer (Invitrogen) at 25 V for 2 hours. The following primary antibodies were used: mouse α-FLAG monoclonal antibody M2 (Sigma Aldrich), rat α-HA 3F10 (Roche Applied Science), mouse α-β-catenin monoclonal antibody C19220 (BD Transduction Laboratories). For detection of proteins, secondary antibodies coupled to HRP (Sigma Aldrich) and ECL Plus Western Blotting Detection Reagents (Amersham Biosciences) were used.

## Results

### Residues of BCL9 and B9L required for the binding to β-catenin

**Figure 1:**
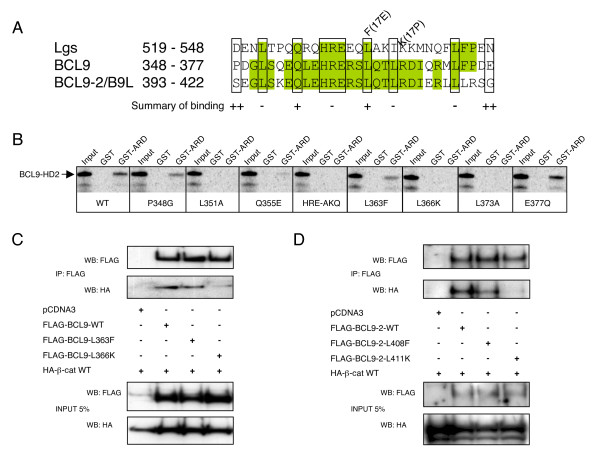
**Mutants of BCL9 proteins that cannot bind to β-catenin**. *A*, Sequence alignments of the HD2 domains of Lgs, BCL9 and BCL9-2/B9L (residue numbers in the latter two refer to mouse or human HD2 sequences, which are identical). Individual conserved residues (*boxed*) were mutated, and semiquantitative estimates of their *in vitro *binding affinities (++/+/-) to the β-catenin ARD are given underneath. *B*, pull-down assays between *in vitro *translated wt and mutant BCL9 HD2 and GST-ARD, as indicated; input lanes, 5% of the total binding reaction. *C and D*, co-immunoprecipitations of FLAG-tagged wt and mutant BCL9 or BCL9-2 with HA-β-catenin expressed in HEK 293T cells; *bottom panels*, protein expression levels in the lysates.

The homology domain 2 (HD2) of BCL9 proteins mediates their binding to Armadillo/β-catenin *in vitro *and *in vivo *[20,24,25,32]. To identify relevant residues within HD2 of human BCL9, we mutagenized amino acids conserved between BCL9, BCL9-2/B9L and Lgs (Fig. 1A), and tested the ability of these mutants to bind the Armadillo repeat domain (ARD) of β-catenin. Additionally, we generated two mutants (L363F, L366K) that mimic loss-of-function alleles of *Drosophila lgs *(*lgs*^17*E*^, *lgs*^17*P*^; Fig. 1A). Using pull-down assays, we found that each of these mutants reduced the binding of HD2 to GST-ARD either partially, or completely (Fig. 1B). In particular, binding was eliminated by L366K, consistent with the results for Lgs [20], but only reduced by L363F (Fig. 1B). Binding was not affected by mutations in residues flanking HD2 (P348G, E377Q; Fig. 1B). We conclude that the three leucine residues 351, 366, 373 and the HRE cluster 358–360 are critical for the binding between BCL9 and β-catenin, consistent with the recent structural analysis [40]. For the following experiments, we decided to use L363F (L>F) as a partial, and L366K (L>K) as a complete loss-of-function mutant.

To test the binding of these mutants to β-catenin *in vivo*, we introduced them into full-length FLAG-tagged BCL9, and also generated the corresponding mutants in BCL9-2 (L408F, L411K; Fig. 1A), and co-expressed these with HA-tagged β-catenin in HEK 293T cells. As expected, HA-β-catenin readily co-immunoprecipitated with wt BCL9, and also with L363F, but not with L366K (Fig. 1C). Likewise, HA-β-catenin co-immunoprecipitated with BCL9-2 and L408F, but not with L411K (Fig. 1D). The converse experiments (immunoprecipitations of HA-β-catenin) showed the same results (not shown). This is consistent with our *in vitro *binding data (Fig. 1B) and confirms that the L366 and L411 residues in BCL9 and BCL9-2, respectively, are critical for their binding to β-catenin (though the L>F substitutions at L363 and L408 are compatible with normal β-catenin binding, at least at high expression levels). Importantly, these results indicate that HD2 is the only domain in BCL9 and BCL9-2 that binds to β-catenin *in vivo*.

### Different activities of overexpressed BCL9 and BCL9-2 in colorectal cancer cell lines

**Figure 2:**
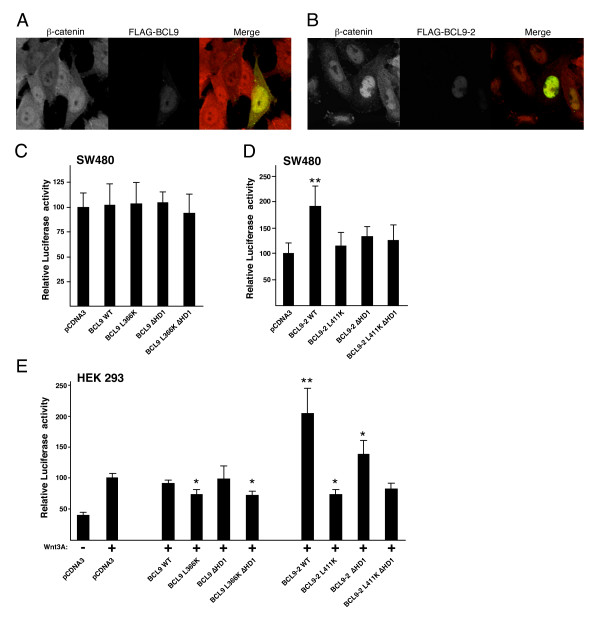
**Subcellular distribution and transactivation potential of wt and mutant BCL9 and B9L**. *A and B*, SW480 cells overexpressing FLAG-BCL9 or FLAG-BCL9-2, fixed and stained with antibodies against β-catenin and FLAG, as indicated in panels (the staining pattern of GFP-B9L was similar to that of FLAG-BCL9-2; not shown). *C-E*, TOPFLASH assays in SW480 and Wnt-stimulated HEK 293 cells (as indicated in panels), expressing wt or mutant FLAG-BCL9 or FLAG-BCL9-2 (see text). Control FOPFLASH assays were also conducted, but the values were less than 5% of TOPFLASH values and did not vary significantly between samples (not shown). Standard deviations are given by bars, and statistical significance relative to values from cells transfected with pCDNA3 (arbitrarily set to 100) is indicated above bars (*, p < 0.05; **, p < 0.001).

To see whether the L>F and L>K mutations affected the subcellular distributions of BCL9 or BCL9-2, we expressed these proteins in SW480 colorectal cancer cells whose Wnt pathway activity is high, due to mutation of APC [41]. As expected from previous studies in other mammalian cells [24,25], BCL9 was distributed throughout the cytoplasm and nucleus (Fig. 2A) while BCL9-2 was strictly nuclear (Fig. 2B; the subcellular distribution of B9L was indistinguishable from that of BCL9-2; not shown). Both proteins showed a tendency to form puncta, however, in neither case could we detect significant differences in the subcellular distributions between wt and mutants (not shown). We also used photobleaching experiments (essentially as described [34,42]) to confirm that GFP-tagged BCL9(HD1+2) is a highly dynamic nuclear-cytoplasmic shuttling protein, like Lgs [34,42], but we were unable to detect any significant differences in the shuttling rates between wt and mutant proteins (not shown).

Given the adaptor role of Lgs/BCL9 [20], full activity would require both an intact HD2 and Pygo-binding domain (called homology domain 1, or HD1). To test this, we overexpressed the L366K and L411K mutants, and BCL9 and BCL9-2 mutants with small internal HD1 deletions (ΔHD1), as well as ΔHD1 L366K and ΔHD1 L411K double-mutants in colorectal cancer cells, and we examined their effects on the Wnt pathway activity of these cells by using TCF reporter activity (TOPFLASH) as a specific and quantitative read-out [39]. We expected one of three outcomes for these ligand-binding mutants: namely, a mutant could be (1) less active than its wt counterpart, implying that the corresponding ligand is required for the function of the wt protein; (2) dominant-negative (DN), suggesting that the mutant protein sequesters a ligand required for the function of the wt protein; or (3) as active as the wt, indicating that the corresponding ligand is not required for the function of the wt protein, at least after overexpression. In particular, we were interested in the behaviour of the double-mutants which cannot bind to either of their known ligands, β-catenin or Pygo: if these were DN, then this would suggest an additional functional ligand of BCL9 proteins.

In SW480 cells, we found that overexpression of wt BCL9 did not affect TOPFLASH activity (Fig. 2C), consistent with previous results of BCL9 overexpression in Wnt-stimulated HEK 293 cells [24]. Likewise, we could not detect any DN effects with any of the BCL9 mutants (Fig. 2C), which were expressed at similarly high levels as the wt (not shown), but this is most likely due to the relatively low transfection efficiency of these cells (see also below, for the behaviour of these mutants in HEK 293 cells whose transfection efficiency is higher). In contrast, overexpressed wt BCL9-2 potentiated TCF reporter activity approximately two-fold in SW480 cells (Fig. 2D), consistent with previous results with B9L [24]. However, none of the BCL9-2 mutants displayed any significant stimulatory activity (Fig. 2D). This indicates that the function of BCL9-2 in TCF-mediated transcription of these cells depends on its ability to bind to β-catenin and Pygo proteins.

We also tested wt and mutant BCL9 proteins in HEK 293 cells transiently stimulated with Wnt3A, since this provides a more sensitive assay (partly due to the higher transfection efficiency of these cells). We optimized this assay, minimizing the Wnt exposure time as much as possible, which allowed us to focus on the primary transcriptional Wnt response and to avoid secondary knock-on effects. We thus found that culturing 293 cells in the presence of Wnt3A for 6 hours resulted in a 2× increase in TOPFLASH activity (Fig. 2E). Like in SW480 cells, overexpression of wt BCL9 did not further increase this activity (Fig. 2E). However, overexpression of L366K, but not of ΔHD1, showed consistently a slight DN effect in reducing TOPFLASH activity compared to the control (Fig. 2E). This DN effect did not appear to be due to the sequestration of Pygo, since it was still detectable with the overexpressed double-mutant L366K ΔHD1 (Fig. 2E). This suggests that this double-mutant BCL9 may sequester a functionally important ligand other than β-catenin and Pygo (see below).

As in SW480 cells, overexpression of BCL9-2 in Wnt3A-stimulated HEK 293 cells resulted in a 2× increase of TOPFLASH activity (Fig. 2E). Its ΔHD1 mutant version also stimulated the TOPFLASH reporter to some extent (by ~50%), whereas L411K showed a slight DN effect, like its L366K counterpart (Fig. 2E), although the ΔHD1 L411K double-mutant appeared neutral and inactive (Fig. 2E). It thus seemed that the latter, unlike its BCL9 counterpart, failed to behave as a DN, but this may have been due to technical reasons (e.g. the expression levels of ΔHD1 L411K were significantly lower than those of L366K ΔHD1; not shown; see also Fig. 1C, D). Taken together, these results indicated that both BCL9 and BCL9-2 rely on their binding to β-catenin and Pygo, and possibly on an additional unknown ligand (at least in the case of BCL9), for their function in mediating Wnt-dependent transcription.

### BCL9 and B9L are Wnt target genes

**Figure 3:**
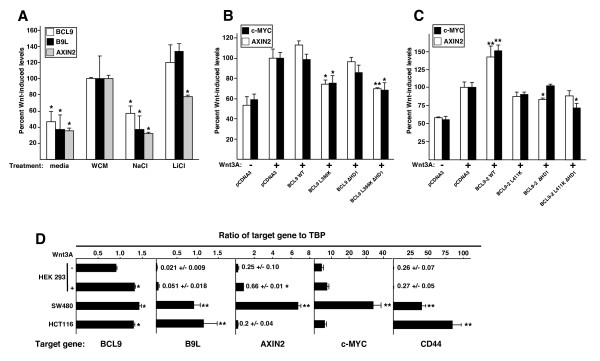
**BCL9 and B9L are Wnt-inducible genes**. *A*, Transcript levels of BCL9 and B9L in comparison to AXIN2, as measured by RT-qPCR, after induction of Wnt pathway activity in HEK 293 cells by addition of Wnt3A-conditioned medium, or 20 mM LiCl, for 6 hours. Statistical significance (p < 0.01) relative to Wnt-stimulated cells (arbitrarily set to 100) is indicated by asterisks. *B and C*, Transcript levels of *c-myc *and *AXIN2 *in Wnt-stimulated HEK 293 cells, measured by RT-qPCR as in *A*, after overexpression of wt or mutant FLAG-BCL9, or FLAG-BCL9-2. Statistical significance relative to Wnt-stimulated cells (arbitrarily set to 100) is indicated by asterisks (*, p < 0.05; **, p < 0.005). *D*, Transcript levels of *BCL9*, *B9L*, *AXIN2*, *c-myc *and *CD44 *relative to *TBP *(as internal control) in HEK 293 (with or without Wnt stimulation), SW480 or HCT116 cells. Statistical significance relative to uninduced HEK 293 cells is indicated by asterisks (*, p < 0.01; **, p < 0.001).

We also tested the effects of overexpressed wt and mutant BCL9 proteins on the Wnt inducibility of endogenous TCF-mediated transcription. Two of the best-established TCF target genes in intestinal epithelial and colorectal cancer cells are *c-myc *and *AXIN2 *[14,16-19]. We stimulated HEK 293 cells for 6 hours with either Wnt3A-conditioned medium, or with LiCl (an inhibitor of GSK3β), and we monitored the transcript levels of these genes by RT-qPCR. As internal controls, we measured the expression levels of TATA-box binding protein (TBP), β-actin and the house-keeping gene *HPRT*. As expected, we observed a 2–2.5× upregulation of *c-myc *and *AXIN2 *transcripts in response to both Wnt3A and LiCl stimulation (Fig. 3A–C), while the transcript levels of the three internal control genes did not change after these treatments. Interestingly, we also discovered that the transcript levels of both *BCL9 *and *B9L *were upregulated to a similar degree (~2× and ~2.5×, respectively) after Wnt stimulation (Fig. 3A). Therefore, *BCL9 *and *B9L *are themselves Wnt-responsive, and are thus likely to represent TCF target genes.

Next, we tested the effects of overexpressed wt and mutant BCL9 proteins on the Wnt-induced expression levels of *c-myc *and *AXIN2*. As expected from the TOPFLASH assays (Fig. 2E), overexpressed wt BCL9 did not stimulate the Wnt-induced expression of these genes further (Fig. 3B), however overexpressed BCL9-2 synergized with Wnt3A to increase the transcript levels of *AXIN2 *and *c-myc *by ~30% (Fig. 3C). Likewise, the BCL9 mutants behaved the same as in the TOPFLASH assays (Fig. 2E), with L366K and L366K ΔHD1 displaying clear DN effects (Fig. 3B). In the case of BCL9-2, each mutant was inactive (i.e. no synergy with Wnt stimulation was observed), and we detected slight albeit statistically significant DN effects of ΔHD1 and L411K ΔHD1 for one target gene each (Fig. 3C). Once again, the DN effect of the double-mutant suggested that BCL9-2, like BCL9, may have an additional functionally important ligand. Taken together, these results fully confirmed those from the TOPFLASH assays, and suggested that the functions of BCL9 proteins regarding the transcriptional Wnt response of endogenous target genes are mediated by Pygo and β-catenin, and possibly also involve a third ligand (see below).

Given the Wnt-responsiveness of *BCL9 *and *B9L *in HEK 293 cells, we wondered whether these genes might be hyperexpressed in colorectal cancer cell lines. We thus used RT-qPCR to monitor their transcript levels in SW480 cells, and also in HCT116 cells whose elevated Wnt pathway activity is due to an activating mutation in β-catenin [41]. We found that the *BCL9 *transcript levels were comparable in both cell lines to those in Wnt-stimulated HEK 293 cells, whereas the *B9L *transcript levels were considerably higher in the colorectal cell lines (35× and 50× higher in SW480 and HCT116 cells, respectively, compared to unstimulated HEK 293 cells; Fig. 3D). These elevated *B9L *transcript levels are similar to those of *BCL9 *in the same cell lines (Fig. 3D). As a comparison, we also measured the expression levels of *AXIN2 *and *c-myc*, and of *CD44 *whose expression in the intestinal epithelium is controlled by TCF and APC [15]. As expected, all three genes are highly expressed in SW480 cells (25×, 6× and ~100× higher, respectively, than in unstimulated HEK 293 cells; Fig. 3D), although in HCT116 cells, only *CD44 *is overexpressed, while the transcript levels of *AXIN2 *and *c-MYC *are lower than in Wnt-stimulated 293 cells (Fig. 3D), in the case of *AXIN2 *due to epigenetic silencing by DNA methylation which has been observed in colorectal cancer cell lines with microsatellite instability, such as HCT116 [43]. Thus, *B9L *and to some extent *BCL9 *are hyperexpressed in colorectal cancer cells compared to unstimulated HEK 293 cells – in the case of *B9L*, more than an order of magnitude, much like some of the other TCF target genes.

### Both BCL9 and B9L are required for Wnt pathway activity

BCL9-2/B9L has previously been shown to be a positive regulator of Wnt signaling in Wnt-stimulated mammalian cells, and in SW480 colorectal cancer cells [24,25]. This is consistent with the DN behaviour of its mutant version, L411K, which cannot bind to β-catenin (Fig. 1D). Given that the corresponding mutant of BCL9 (L366K) showed similar DN effects (at least in Wnt-stimulated HEK 293 cells), we asked whether BCL9 is also required for efficient Wnt pathway activity in SW480 cells.

**Figure 4:**
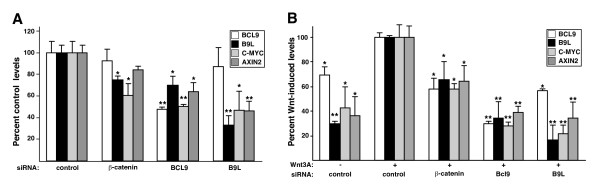
**BCL9 is required for the transcription of endogenous Wnt target genes**. Transcript levels of *BCL9*, *B9L*, *c-MYC *and *AXIN2*, as measured by RT-qPCR, in (*A*) SW480 cells or (*B*) Wnt-induced HEK 293 cells (as in Fig. 3), treated with siRNA to deplete β-catenin, BCL9 or B9L. Statistical significance (*, p < 0.01 and **, p < 0.001) relative to controls (set arbitrarily to 100%) is indicated above bars.

We thus used siRNA-mediated depletion to test this, and also to assess the relative contributions of BCL9 and B9L to TCF-mediated transcription in SW480 cells. This allowed us to reduce the transcript levels of BCL9 to ~50%, and those of B9L to ~30% of their normal levels in these cells (Fig. 4A); under the same conditions, we were able to deplete the levels of β-catenin transcripts to ~25% (not shown). We found that the expression levels of *c-myc *and *AXIN2 *were reduced after depletion of *BCL9 *to a similar degree as after depletion of *B9L*, or after depletion of β-catenin transcripts (Fig. 4A). *B9L *expression was also reduced after *BCL9 *depletion (which thus mimics a double knock-down of both paralogs, albeit a partial one) although the converse was not true, possibly because this gene is only mildly Wnt-inducible, and is less hyperactive than *B9L *in colorectal cancer cell lines (see Fig. 3). These results indicate that BCL9 is required for efficient TCF-mediated transcription in these colorectal cancer cells, similarly to B9L and β-catenin.

We also depleted *BCL9 *transcripts in Wnt-stimulated HEK 293 cells, to test the role of BCL9 in TCF-mediated transcription under transient Wnt signaling conditions. Again, we found marked reductions of *c-myc*, *AXIN2 *and *B9L *after *BCL9 *depletion, similarly as after depletion of *B9L *(Fig. 4B). Indeed, the effects on Wnt target gene expression appeared stronger in these cells, most likely due to their higher transfection efficiency (i.e. a higher fraction of cells experienced RNAi-mediated depletion). We conclude that BCL9, like B9L, is a positive regulator of Wnt-induced transcription in HEK 293 cells.

### The C-termini of BCL9 proteins are required for their function in Wnt signaling

Previous work revealed a DN effect of a C-terminal deletion of B9L in colorectal cancer cells, suggesting a role of the C-terminus of B9L in its Wnt response [24]. Likewise, our result that the BCL9 double-mutant behaved as a DN, despite being defective in Pygo and β-catenin binding (see above), suggested that this protein may bind to an additional ligand required for its function in Wnt signaling. We thus generated a C-terminal deletion (ΔC) of BCL9, to test whether this would behave as a DN in Wnt-stimulated HEK 293 cells. Furthermore, we introduced the L366K and ΔHD1 single and double mutations into this C-terminal truncation, and also into the ΔC truncation of B9L [24], to ask whether the DN effect(s) would be abolished if β-catenin and/or Pygo binding was eliminated.

**Figure 5:**
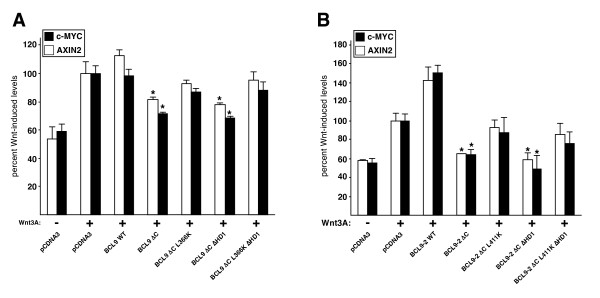
**Requirement of the C-terminus of BCL9 for Wnt-induced transcription**. Transcript levels of *c-MYC *and *AXIN2*, as measured by RT-qPCR, in Wnt-induced HEK 293 cells (as in Fig. 3) after overexpression of wt or mutant (*A*) FLAG-BCL9 or (*B*) FLAG-BCL9-2. Statistical significance (p < 0.01) relative to Wnt-stimulated control cells (set arbitrarily to 100%) is indicated by asterisks above bars.

Indeed, the ΔC mutant of BCL9 showed a mild DN effect on the TOPFLASH activity of Wnt-stimulated HEK 293 cells (Fig. 5A). By comparison, B9L ΔC behaved as a more potent DN [24], reducing the TOPFLASH values almost to those measured in unstimulated cells (Fig. 5B), possibly because it could compete more effectively with endogenous B9L (whose expression level is far lower than that of BCL9; Fig. 3D) and is targeted to the nucleus more efficiently than BCL9 (Fig. 2A, B). Interestingly though, in both cases, the DN effects remained detectable in the ΔC ΔHD1 double-mutants, but was eliminated in the ΔC L366K/L411K double-mutants, and in the triple-mutants (Fig. 5A,B), all of which are defective in β-catenin binding. These results indicated that the C-terminus of BCL9, like that of B9L, harbours a function in TCF-mediated transcription, thus explaining why the ΔHD1 L366K and ΔHD1 L411K double-mutants behave as DNs (Fig. 2E; 3B, C). Furthermore, the results from all three types of double-mutants imply that the putative sequestration of β-catenin (by ΔC ΔHD1) or C-terminal ligand (by ΔHD1 L366K and ΔHD1 L411K) reduced Wnt-mediated transcription, whereas sequestration of Pygo (by ΔC L366K and ΔC L411K) did not affect this process.

## Discussion

### The function of BCL9 and B9L depends on their binding to β-catenin

Our mutational analysis of HD2 revealed multiple residues in this domain that are critical for β-catenin binding *in vitro*, including three leucine residues whose mutation ablated binding. This is fully consistent with recent structural data: HD2 forms an α-helix which contacts the N-terminal end of the ARD of β-catenin, whereby two of these leucines (L366, L373) are located at the interface between the two domains and are engaged in direct contacts with β-catenin (the third, L351, was not visible in the structure) [40]. Importantly, the individual L>K mutations which abolished β-catenin binding *in vitro *also eliminated association of BCL9 and B9L with β-catenin *in vivo*, so HD2 is the only domain through which these proteins can bind to β-catenin in cells. Consistent with this, the corresponding L>K mutation in *Drosophila *(in the *lgs*^17*P *^allele) results in Lgs loss-of-function, and loss of Armadillo binding [20]. Somewhat puzzlingly, the L>F mutants reduced β-catenin binding of BCL9 proteins only mildly *in vitro*, and not detectably *in vivo *(Fig. 1), so it is less clear why the corresponding mutation in the *lgs*^17*E *^allele should inactivate Lgs in *Drosophila *development [20].

### BCL9 proteins are necessary for efficient TCF-dependent transcription

The functional link between BCL9 and β-catenin indicated a role for BCL9 in Wnt signaling. Indeed, our loss-of-function analysis by RNAi in Wnt-stimulated HEK 293 and SW480 cells provided strong evidence for this, revealing that BCL9 is required for efficient TCF-mediated reporter gene transcription and for Wnt-inducibility of endogenous TCF target genes. Therefore, BCL9 is a positive regulator of the Wnt pathway in human cells, similarly to its relative B9L [24,25], and to its *Lgs *counterpart in *Drosophila *[20].

We confirmed earlier findings that BCL9 exhibits a subcellular distribution distinct from that of B9L/BCL9-2 [24,25]: BCL9 was found throughout the cytoplasm and nucleus after overexpression, whereas B9L was entirely nuclear (Fig. 2A, B). This constitutive nuclear location of B9L is due to a nuclear localization sequence (NLS) in its N-terminus [25]. In contrast, BCL9 is a 'conditionally nuclear' protein that can shuttle in and out of the nucleus, like Lgs [34,42]. Indeed, the nuclear location of Lgs depends entirely on its binding to Pygo [34], and the same may also be the case for BCL9. In turn, Pygo proteins are constitutively nuclear by virtue of their own NLS (e.g. [20,21,31,34,44]), and Pygo-BCL9/Lgs complexes can bind to methylated histone H3 tail through the PHD fingers of Pygo [45]. Notably, in *Drosophila*, Pygo is associated with dTCF target genes even in the absence of Wingless signaling [35]. Pygo proteins thus appear to be nuclear retention factors for the conditionally nuclear BCL9 proteins, and may recruit them to TCF target genes. It is unclear why vertebrates have also evolved a constitutively nuclear version of BCL9/Lgs. Given the Wnt-inducibility of *B9L *in human cells and its overexpression in colorectal cancer cells, it is conceivable that the constitutively nuclear B9L/BCL9-2 proteins serve to maintain, or boost, Wnt-mediated transcription in cells whose Wnt pathway is persistently active (see below).

The distinct subcellular locations of BCL9 and B9L may explain why only B9L, but not BCL9, is able to potentiate Wnt-induced transcription when overexpressed in HEK 293 and SW480 cells (Fig. 2D, 3B, C; Fig. 5). It is likely that the nuclear retention factors of BCL9 – e.g. the Pygo proteins – are limiting under these conditions, especially since endogenous BCL9 is also expressed at comparatively high levels in both cell types (Fig. 3D). Note that, although the constitutive nuclear location of B9L is likely to be independent of Pygo, its function nevertheless relied on its interaction with Pygo, given that the ΔHD1 mutant of B9L was unable to stimulate Wnt-mediated transcription (Fig. 2D, 3C).

Apart from their distinct subcellular distributions and overexpression phenotypes, BCL9 and B9L appear to be equivalent regarding their roles in Wnt signaling. In particular, their *in vivo *associations with β-catenin were comparable (Fig. 1), probably reflecting similar binding affinities, given that their β-catenin-binding residues in HD2 are highly conserved [40]. Likewise, the Pygo-binding residues in HD1 are conserved between the two BCL9 proteins [45], implying comparable affinities to Pygo as well. Of course, there may be other differences between BCL9 and B9L with regard to unknown ligands and/or functions (see below).

### Evidence for a functional ligand of the C-termini of BCL9 proteins

Two pieces of evidence indicated an additional unknown ligand of the BCL9 C-terminus that is critical for the function of BCL9 in Wnt-mediated transcription. Firstly, a BCL9 double-mutant whose binding to β-catenin and Pygo was eliminated behaved as a DN in Wnt signaling assays. This DN effect is unlikely due to direct interference with endogenous BCL9 (and/or B9L) via dimerization, since we were neither able to detect self-association of differently tagged BCL9 proteins, nor heterodimerization between BCL9 and B9L, in transfected HEK 293 cells; lack of interaction between the two proteins was also apparent in sensitive *in vivo *recruitment assays (i.e. B9L was unable to recruit BCL9 into the nucleus on co-expression; J. W., unpublished data). Secondly, a C-terminal truncation of BCL9 also behaved as a DN in Wnt-stimulated mammalian cells, like the equivalent truncation of B9L [24]. This was somewhat surprising, given that an equivalent truncation of Lgs was able to rescue *lgs *mutants in *Drosophila *[20]. However, it is worth noting that these rescue assays involved overexpression, and therefore possibly masked a function of the Lgs C-terminus. Indeed, 1 of the 6 known *lgs *mutations results in a similar C-terminal truncation [20], indicating that the C-terminus of Lgs does in fact have an important function during normal development in *Drosophila*.

Evidence for an additional ligand of BCL9 proteins with a function in Wnt signaling was also provided by the double-mutants whose binding to Pygo and β-catenin was eliminated: these behaved as DNs, possibly by sequestering a functionally important ligand. If so, and if this third ligand bound to the C-terminus of BCL9 proteins, then the DN effects of the double-mutants should be eliminated in triple-mutants that also lack the C-terminus, which was indeed the case. It thus appears that the triple-mutants are no longer able to sequester any functional ligand, supporting the notion that BCL9 proteins have three rather than two functionally important ligands. Our evidence for a third C-terminal ligand of BCL9 proteins argues against the simple adaptor hypothesis as initially proposed, which envisaged that the sole function of BCL9 proteins was to link Pygo and Armadillo/β-catenin [20].

During the final stages of manuscript preparations, a study was published that uncovered a cell-type specific function of BCL9 in TCF-responsive transcription in B cell lines [46]. This function appears to be independent of Pygo proteins, but reliant on a trans-activation domain in the C-terminus of BCL9. It is conceivable that this domain is responsible for the dominant-negative effects of our C-terminal BCL9 (and B9L) deletions.

### *BCL9 *and *B9L *are Wnt target genes

In the course of our analysis, we discovered that *BCL9 *and *B9L *are both Wnt-inducible genes, and that they are hyperexpressed in colorectal cancer cells whose Wnt pathway is constitutively active. Therefore, both genes are likely to be TCF target genes, much like *c-myc *and *AXIN2 *[14,16,17]. In particular, *B9L *expression was highly responsive to changes in Wnt pathway activity, and its hyperexpression in colorectal cancer cells was pronounced. Notably, high levels of *B9L *transcripts have also been found in 6 of 14 of colorectal tumour samples with elevated Wnt pathway activity [24], and the level of *B9L *overexpression correlated with the progression from benign colorectal adenomas to invasive carcinomas [47].

Our findings suggest that BCL9 and B9L are part of a positive feedback mechanism. In particular, B9L may function to maintain, or boost, TCF-mediated transcription once initiated by the stimulation of cells by extracellular Wnt ligand. Indeed, overexpression of B9L and BCL9 may contribute to, or even confer, persistent pathway activity that is independent of Wnt ligand, for example in colorectal tumours whose progression may thus be promoted by these proteins.

### The potential of BCL9 as a drug target in colorectal cancer

Although the Wnt/β-catenin pathway is an important cancer pathway, there are no well-established, validated small molecule inhibitors of this pathway to date. The likely reason for this is that the cancer-promoting Wnt pathway mutations typically occur at the level of, or immediately above, β-catenin [2,48]. However, there are no known enzymes below activated β-catenin that could be inhibited, and the protein interaction surface between β-catenin and TCF is highly unsuitable for disruption by small molecule inhibitors since it is extensive, and because negative regulators of β-catenin such as Axin and APC bind to the same TCF-interacting surface of β-catenin [49].

Our evidence for the requirement of BCL9 in the TCF-dependent transcription of colorectal cancer cells, and its hyperexpression in these cells, has revealed its potential as a target for inhibitory drugs. Furthermore, the potential for disruption of the binding between β-catenin and BCL9 is indicated by our result that single point mutations in HD2 disrupt the association of β-catenin and BCL9, or B9L, *in vitro *and *in vivo *(Fig. 1), consistent with the structural analysis of the β-catenin-BCL9 complex, which also illustrates the importance of individual residues for their mutual interaction [40]. However, further validation of BCL9 proteins as drug targets in colorectal cancer will be required, including the analysis of their function in Wnt signaling during mammalian development and in adult tissue homeostasis, and of their role in promoting intestinal tumorigenesis in animal models.

## Conclusion

Our loss-of-function analysis of BCL9 in human cell lines has led to the following three conclusions. Firstly, BCL9 is required for efficient TCF-mediated transcription in Wnt-stimulated and colorectal cancer cells. Secondly, this function of BCL9 depends on β-catenin, but also on its C-terminus, implying that this conserved part of the protein binds to an unknown ligand that is important for its Wnt-related function. Thirdly, both *BCL9 *and *B9L *are Wnt-inducible genes that are hyperexpressed in colorectal cancer cell lines, indicating that they are part of a positive feedback loop, reinforcing Wnt pathway activity. The functional importance and hyperexpression of BCL9 in colorectal cancer cells indicates the potential of this protein as a cancer drug target.

## Competing interests

The authors declare that they have no competing interests.

## Authors' contributions

MdlR conducted most of the experiments in this study. JW initiated the study, and contributed Fig. 1C &1D and Fig. 2A &2B. MB was responsible for the overall design of the study, and drafted the manuscript. All authors read and approved the manuscript.

## Pre-publication history

The pre-publication history for this paper can be accessed here:


